# Supplementary material for: Impaired recruitment of dopamine neurons during working memory in mice with striatal D2 receptor overexpression
Source: Nat Commun. 2018 Jul 19;9:2822. doi: 10.1038/s41467-018-05214-4 (PMC6053467; doi:10.1038/s41467-018-05214-4)
Supplement: Supplementary file 1 — b-h are now included in Supplementary Figure 8, (so there is no white space as in the previous version)."?>Supplementary Information [file 41467_2018_5214_MOESM1_ESM.pdf]

Supplementary Information

Impaired recruitment of dopamine neurons during working memory in mice with striatal D2 receptor overexpression

Sevil Duvarci, Eleanor H. Simpson, Gaby Schneider, Eric R. Kandel, Jochen Roeper and Torfi Sigurdsson

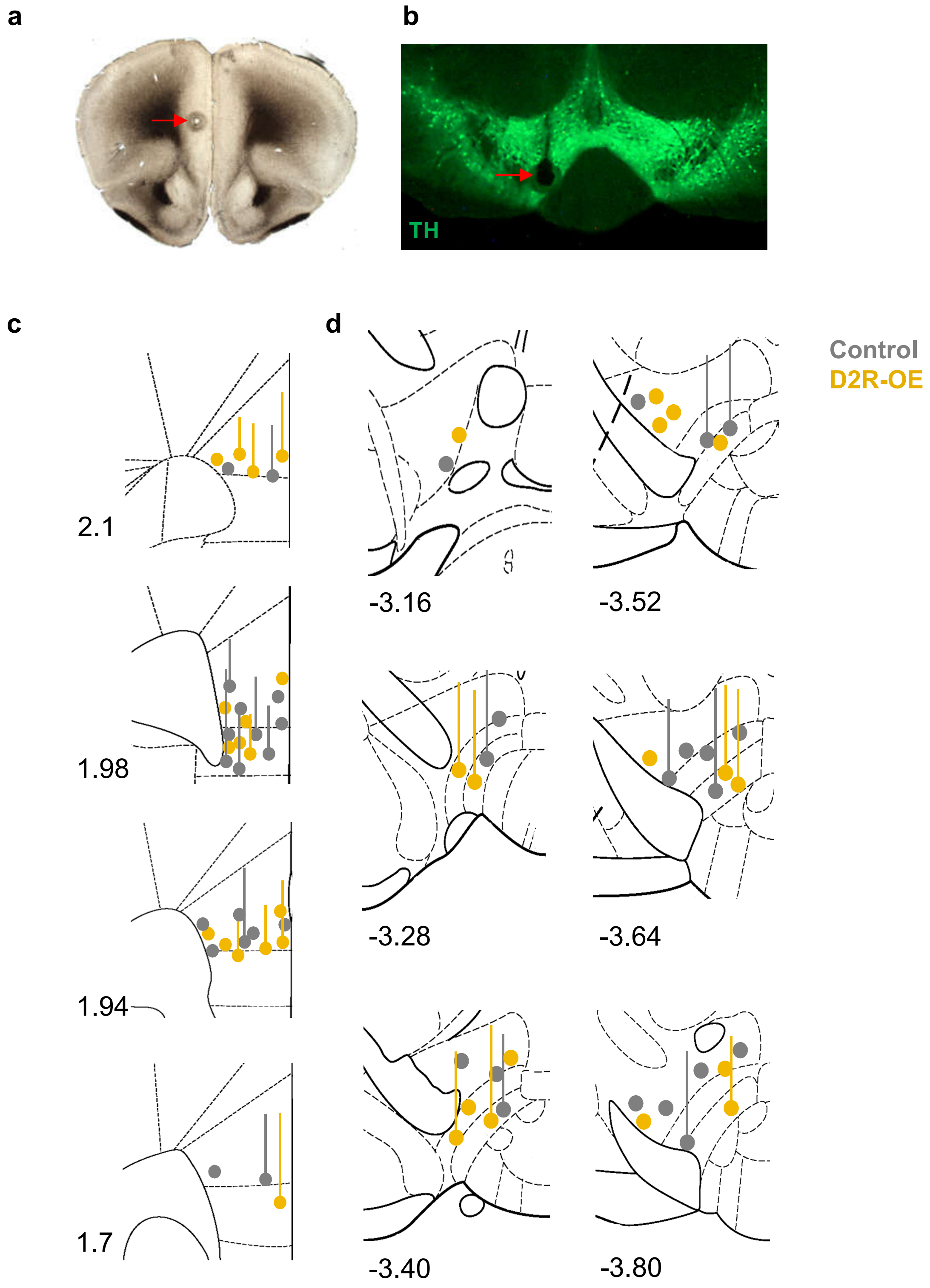

Supplementary Figure 1. Placement of electrodes in VTA and PFC. a,b, Representative brain sections showing electrolytic lesions (red arrows) marking electrode locations in the PFC **(a)** and VTA **(b)**. Green in **(b)** represents staining against tyrosine hydroxylase used to identify the VTA. **c,d,** Schematic of electrode locations in the PFC **(c)** and VTA **(d)** in control (gray) and D2R-OE (orange) mice. Lines represent electrode tracks of moveable stereotrodes; circles without lines indicate location of LFP electrodes. Numbers represent distance in mm anterior (PFC) or posterior (VTA) to bregma. Brain diagrams in c and d are adapted with permission from REF. 62, Elsevier.

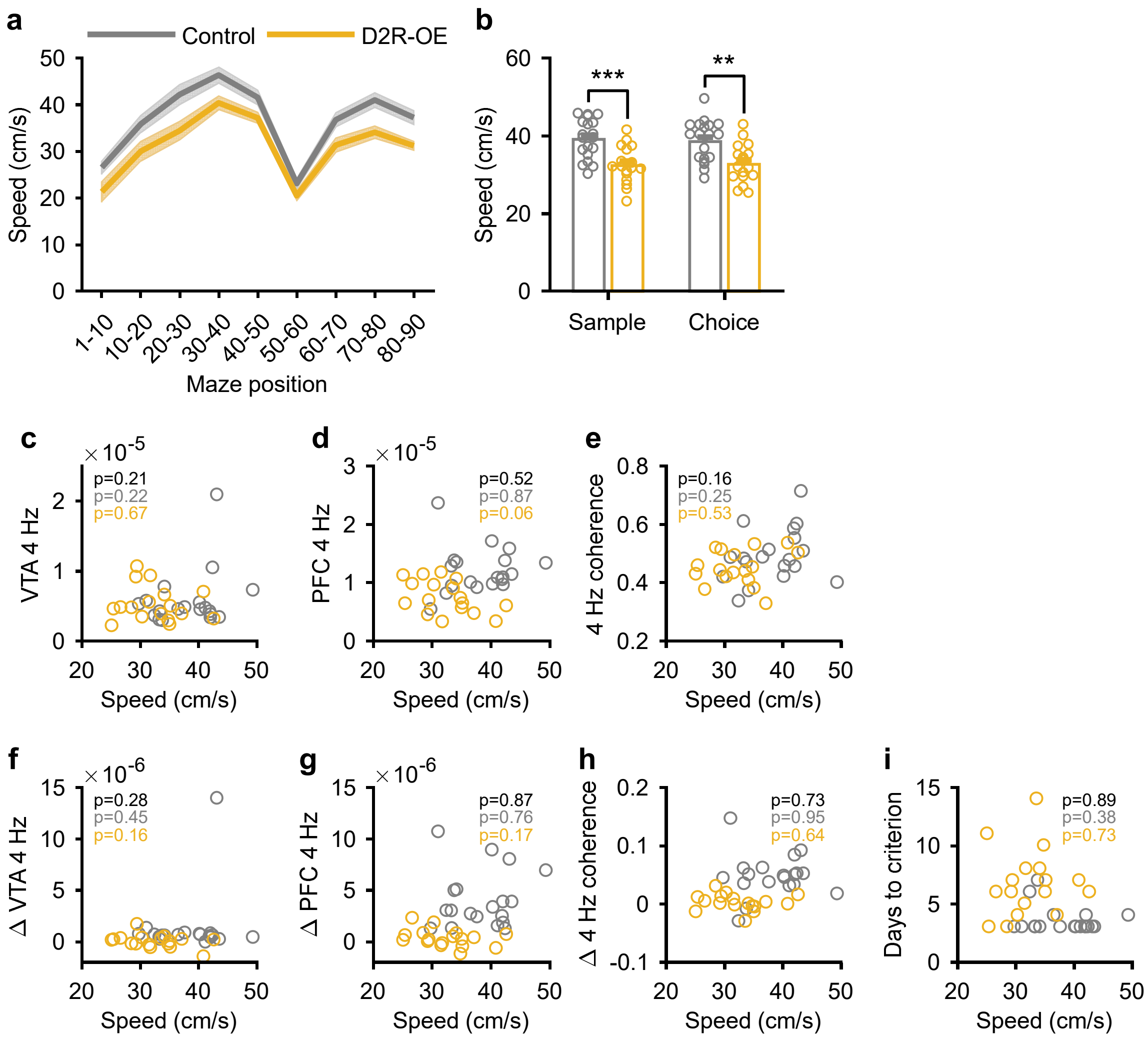

Supplementary Figure 2. Running speed in D2R-OE and control mice and its relationship to neural activity and task performance. **a,** Running speed in the choice phase as a function of linearized maze position. The decrease in running speed corresponds to when animals reach the choice point in the T-maze. **b,** Average running speed in the center arm of the T-maze. Running speed was lower in D2R-OE mice in both sample and choice phases (**$p<.01$;***$p<.001$, rank-sum test). However, running speed did not differ between sample and choice phases for either genotype (Control: $p=0.26$; D2R-OE: $p=0.31$, sign-rank test). **c-e,** Relationship between running speed and VTA 4 Hz power **(c),** PFC 4 Hz power **(d),** and 4 Hz coherence **(e)** in the choice phase. **f-h,** Relationship between running speed in choice phase and choice−sample differences (Δ) in VTA 4 Hz power **(f),** PFC 4 Hz power **(g),** and 4 Hz coherence **(h)**. **i,** Relationship between speed and learning rate. All data points represent average values for each animal. P values indicate the significance of the relationships, assessed using all animals (effect of speed in a genotype X speed ANCOVA; black), control animals alone (Pearson's correlation; gray) or D2R-OE animals alone (Pearson's correlation; yellow). Neither 4 Hz power, coherence or learning rate correlated with running speed. Shaded regions and error bars represent mean±s.e.m measured across animals.

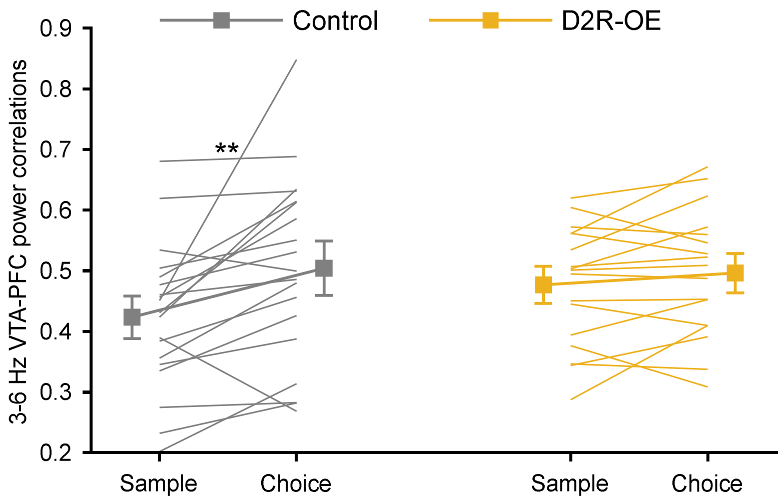

Supplementary Figure 3. Correlations between 4 Hz power in the VTA and PFC during sample and choice phases. Individual thin lines depict the strength of power correlations (Pearson's r) in the two task phases for each animal. The strength of 4 Hz power correlations increased in the choice phase in control animals (**p<.01, sign-rank test), but not in D2R-OE, mice (p=0.27, sign-rank test). Error bars represent mean±s.e.m across animals.

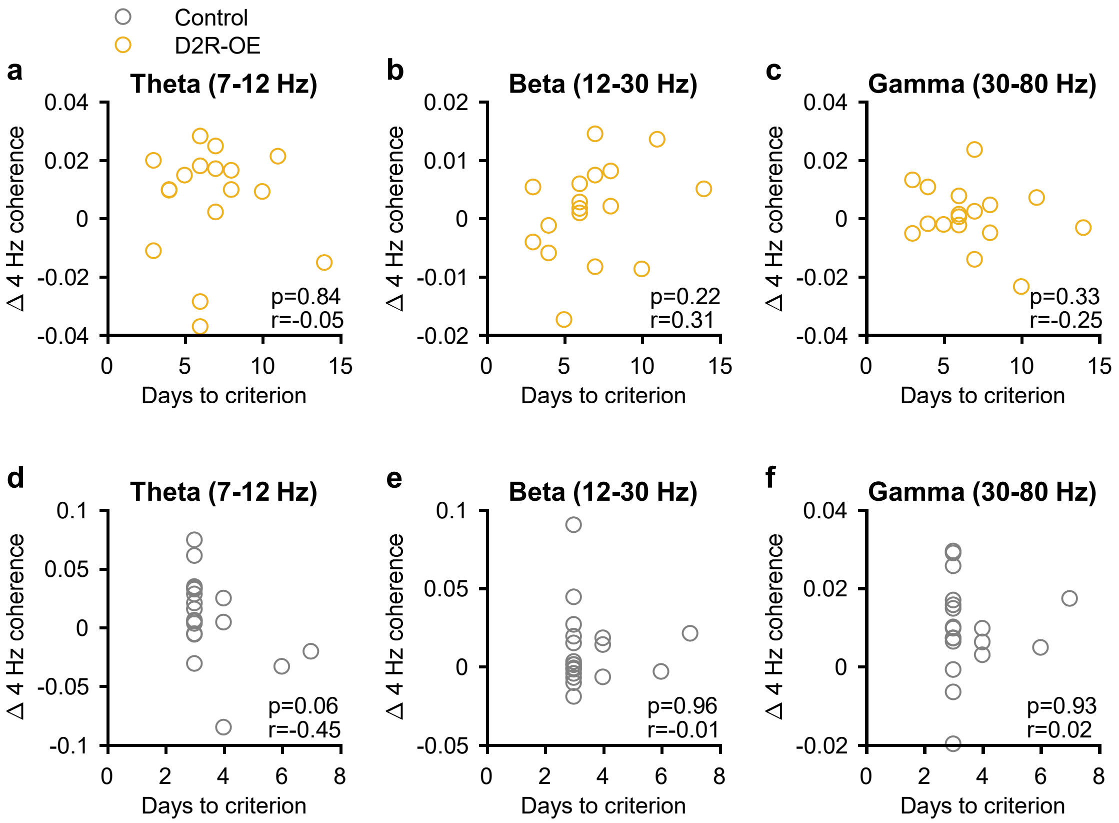

Supplementary Figure 4. Working memory deficits are not correlated with VTA-PFC coherence in the theta, beta or gamma frequency range. Each scatter plot shows, for each animal, its learning rate (days to criterion) versus choice−sample differences (Δ) in VTA-PFC coherence (as in Figure 3e) for three different frequency bands: theta (7-12 Hz), beta (12-30 Hz) and gamma (30-80 Hz). Correlations are shown separately for D2ROE (**a-c**) and control (**d-f**) animals. Numbers in the lower right corner of each scatterplot indicate Pearson's r and its associated p value**.**

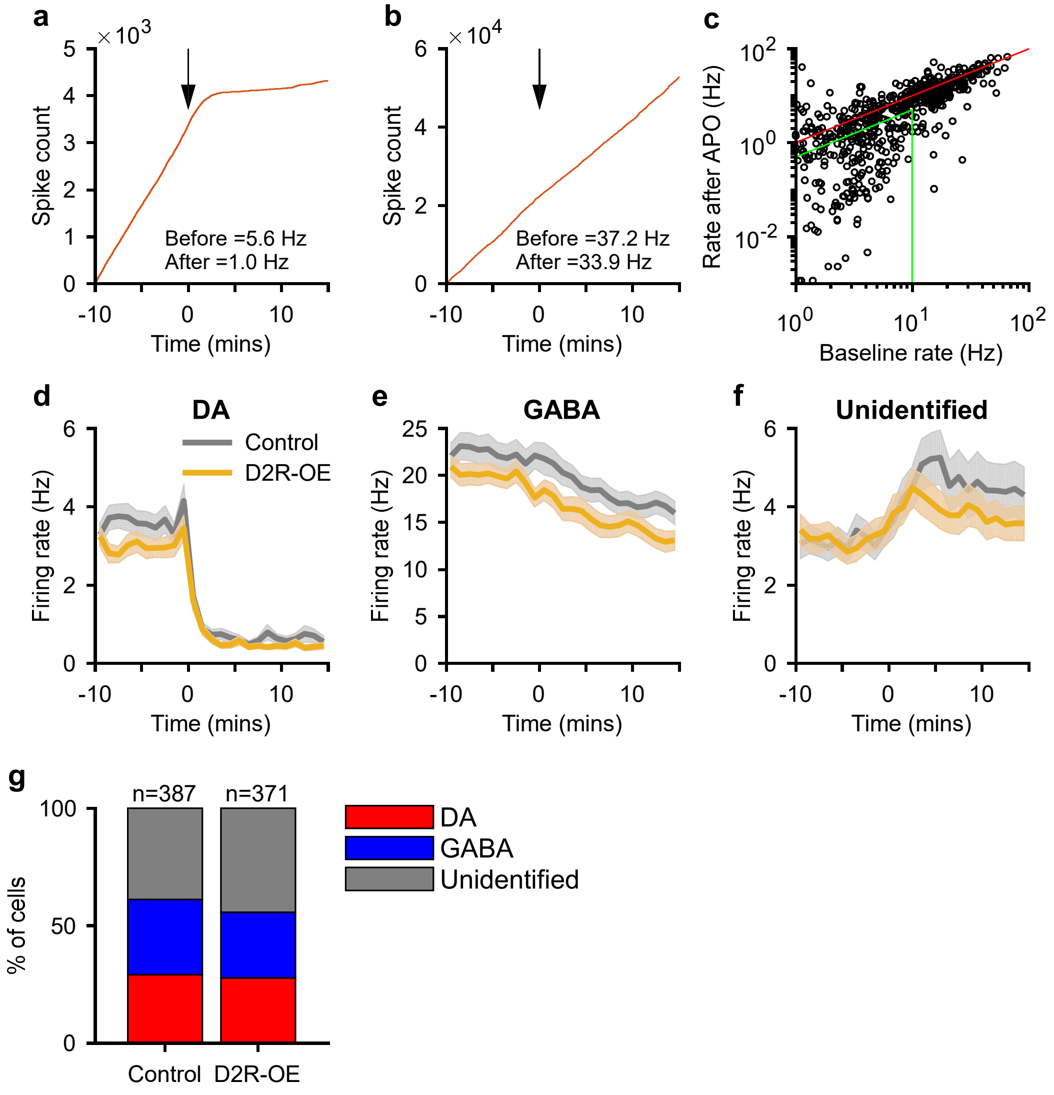

Supplementary Figure 5. Classification of VTA DA and GABA neurons. a,b, Responses of a putative DA **(a)** and GABA **(b)** neuron to apomorphine (APO) injection. Y axis represents the cumulative spike count; the x axis represents time relative to APO injection (arrow). **c,** The baseline firing rate of each VTA neuron (x-axis) is plotted against its firing rate after APO injection (y-axis). The unity line is shown in red. Note the stronger inhibitory effects of APO on neurons with baseline firing rates below 10 Hz. Neurons within the green boundary were classified as putative DA neurons (baseline firing rate < 10 Hz, > 50% reduction in firing rate following APO administration). Neurons with baseline firing rates > 10 Hz were classified as putative GABA neurons. **d-f**, Firing rates before and after APO administration in control and D2R-OE mice for putative DA **(d)**, GABA **(e)** and unidentified **(f)** neurons. **g,** The proportion of putative DA and GABA neurons was similar in control and D2R-OE mice (DA: $p=0.69$, GABA: $p=0.24$, Fisher's exact test). Shaded regions and error bars represent mean±s.e.m measured across neurons.

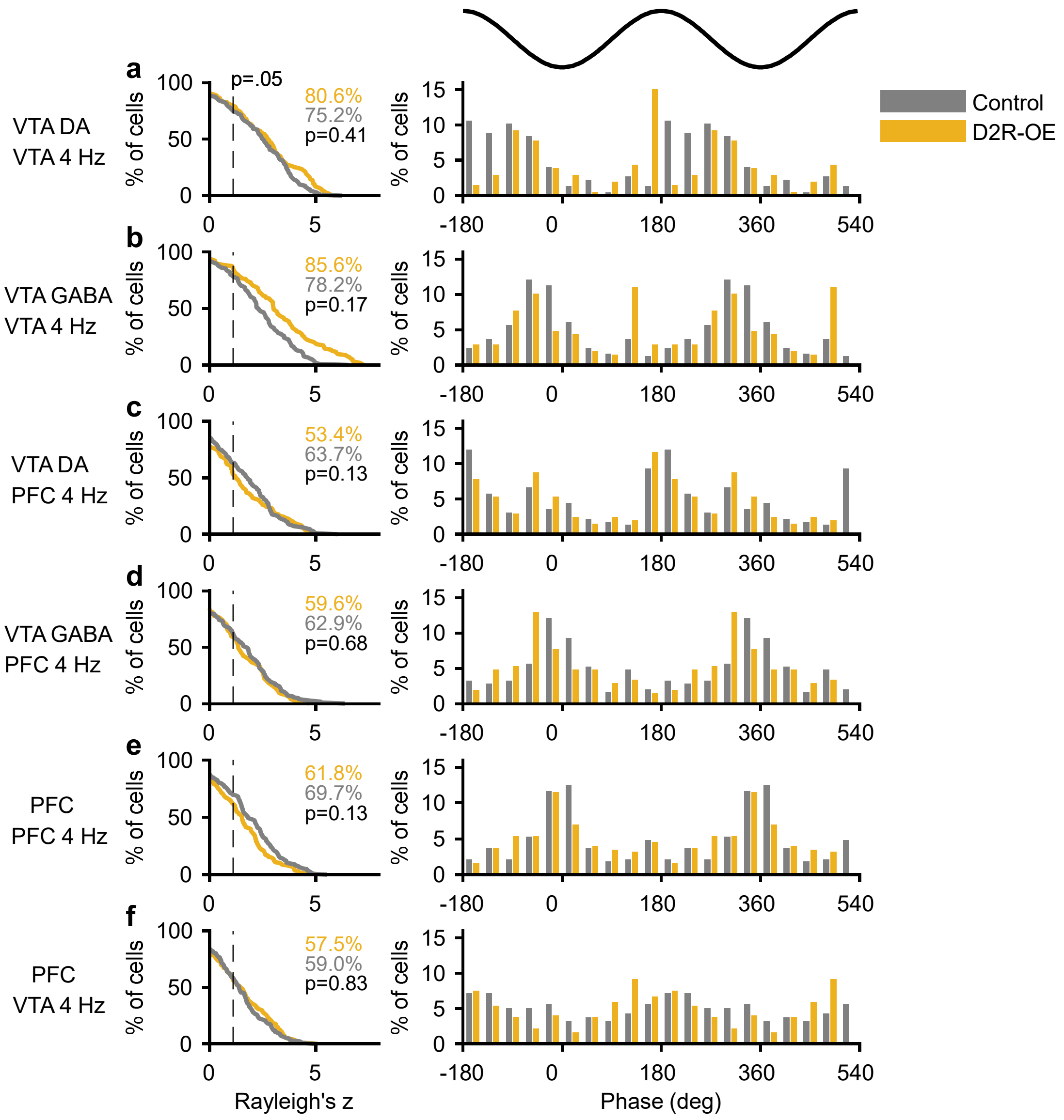

Supplementary Figure 6. Phase-locking of VTA and PFC neurons to 4 Hz oscillations in control and D2R-OE mice. Each row compares the phase-locking of VTA DA **(a,c)** VTA GABA neurons **(b,d)** and PFC neurons **(e,f)** to 4 Hz oscillations in either VTA **(a,b,f)** or PFC **(c,d,e)** between control and D2R-OE mice. Plots on the left show the cumulative distribution of Rayleigh's z scores; dotted line indicates the significance threshold (p=.05). Percentages indicate the proportion of significantly phase-locked cells (p<.05, Rayleigh's test for uniformity) in each genotype and the p values indicate the significance of their differences between genotypes (Fisher's exact test). Bar graphs on the right show the distribution of preferred 4 Hz phases for neurons that are significantly phase-locked to the 4 Hz oscillation. The proportion of neurons phase-locked to 4 Hz oscillations, and their preferred phase of firing, was similar in D2R-OE and control animals.

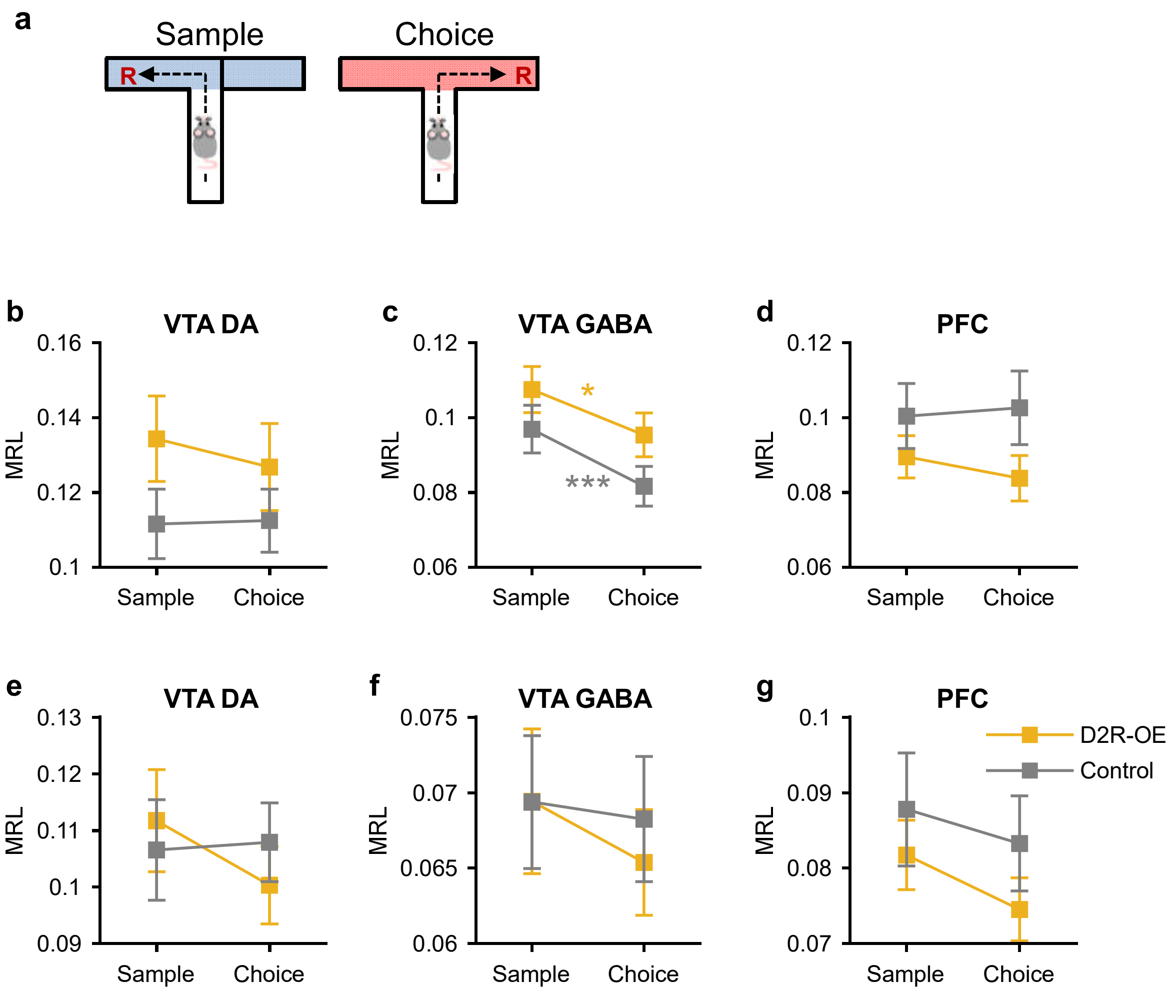

Supplementary Figure 7. Four Hz phase-locking during sample and choice phases in the goal arms of the T maze. a, Phase-locking strength was compared in the sample and choice phases in the goal arms of the T-maze, after animals had made a decision to turn left or right. The strength of phase-locking of VTA DA **(b,e**; Control: n=85, D2R-OE: n=85**)**, VTA GABA **(c,f**; Control: n=142, D2R-OE: n=144**)** and PFC **(d,g**; Control: n=77, D2R-OE: n=119**)** neurons to the 4 Hz oscillation in the VTA **(b-d)** or the PFC **(e-g)** did not increase in the choice phase (all p>0.05, sign-rank test). However, the phase-locking of VTA GABA neurons to VTA 4 Hz oscillations was lower during the choice phase (**c,** ***p<.001, *p<.05, sign-rank test). Thus, the increase in phase-locking during the choice phase is selective to the center arm, where working memory is required. Error bars represent mean±s.e.m measured across neurons.

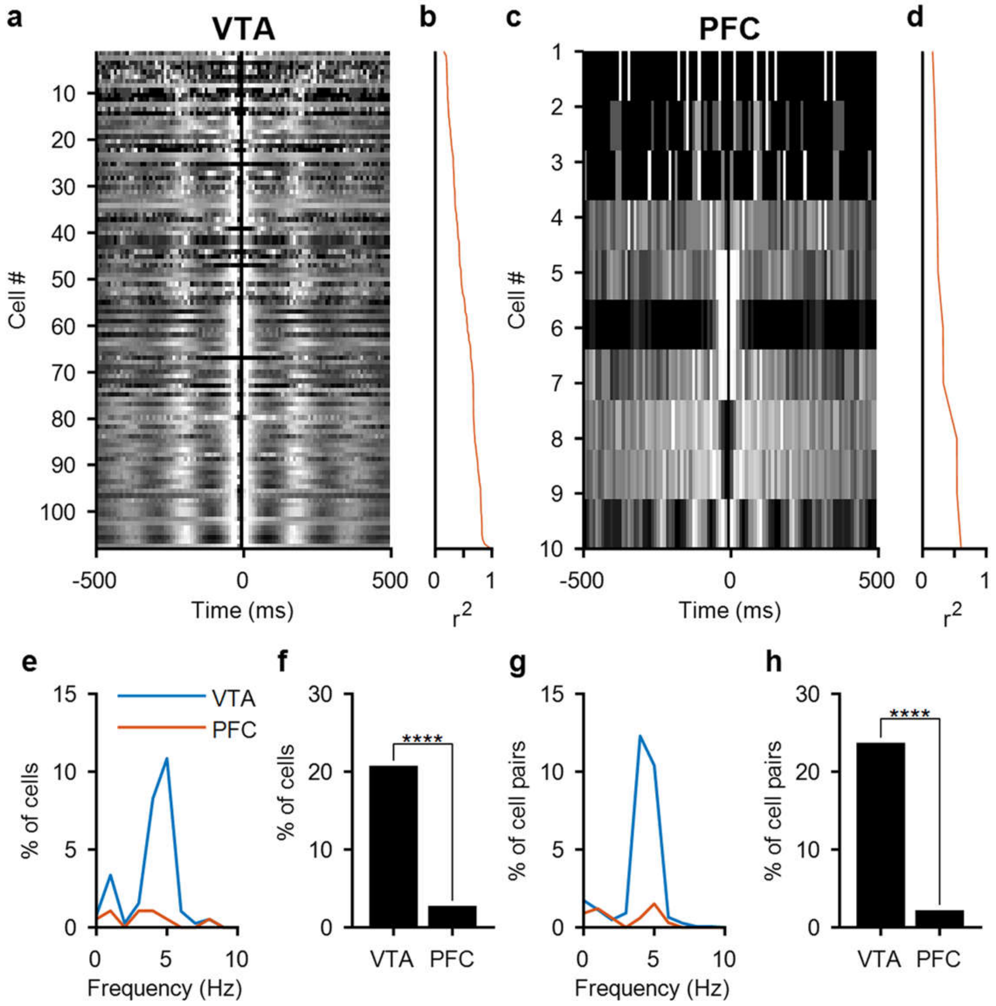

Supplementary Figure 8. Comparison of rhythmic 4 Hz firing in VTA and PFC neurons. a,c, Autocorrelograms of VTA **(a)** and PFC **(c)** neurons showing significant rhythmic firing. Each row represents the autocorrelogram of a single neuron (black=0, white=1). Autocorrelograms are sorted by their goodness of fit (r^2) to the sinusoidal component of the function used to fit the correlograms, which is shown in **b** and **d. e,** Distribution of frequencies at which rhythmic firing was observed for VTA and PFC neurons. **f,** Proportion of VTA (n=387) and PFC (n=188) neurons displaying rhythmic firing in the 4 Hz range (3-6 Hz). **g,** Distribution of frequencies at which PFC and VTA neuron pairs showed coordinated rhythmic firing. **h,** Percentage of VTA (n=1846) and PFC (n=330) neuron pairs displaying rhythmic coordinated firing in the 4 Hz range. Note the relative lack of rhythmic 4 Hz firing in PFC compared to VTA neurons (****p<.0001, Fisher's exact test), suggesting that 4 Hz oscillations are generated in the VTA. Only data from control animals was included in these analyses.

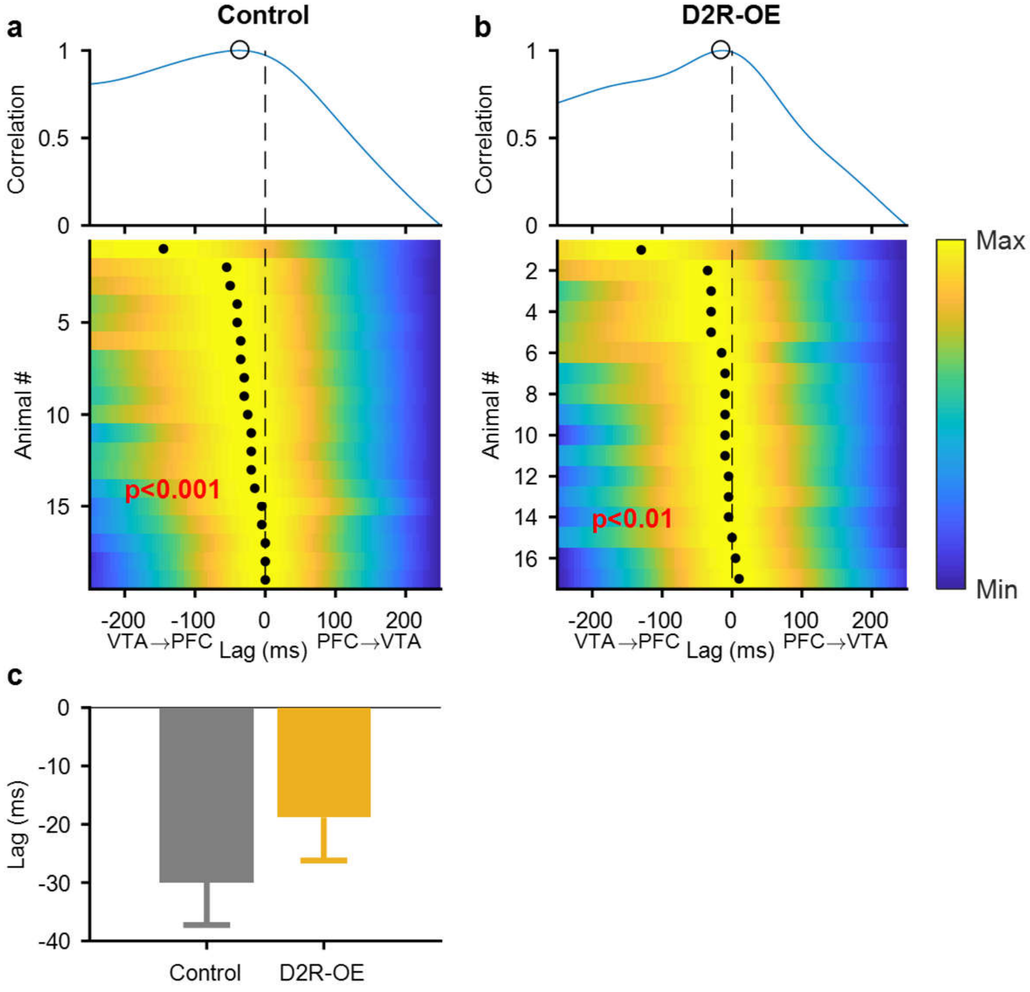

Supplementary Figure 9. Four Hz oscillations in the VTA lead 4 Hz oscillations in the PFC. a,b Cross-correlation of 4 Hz power in the PFC and VTA of control **(a)** and D2R-OE **(b)** mice. Top, example of a cross-correlogram from a single animal. Lag at which correlations were highest is indicated by black circle. Bottom, cross-correlograms of all animals sorted by the lag at which power correlations were largest (indicated by filled black circles). All correlograms are normalized and correlation strength is represented by color (0=minimum correlation, blue; 1=maximum correlation, yellow). Power correlations were strongest at negative lags in both control (n=19, p<.001, sign-rank test) and D2R-OE (n=17, p<.01) mice, indicating that changes in 4 Hz power in the VTA lead changes in the PFC. This is consistent with the possibility that 4 Hz oscillations are generated in the VTA and transmitted to the PFC. **c,** Comparison between cross-correlation lags in the two genotypes. The average lag at which power correlations were maximal did not differ between genotypes (p=0.09, rank-sum test) suggesting that the directionality of influence between the VTA and PFC is not altered in D2R-OE mice. Error bars represent mean ± s.e.m over animals.

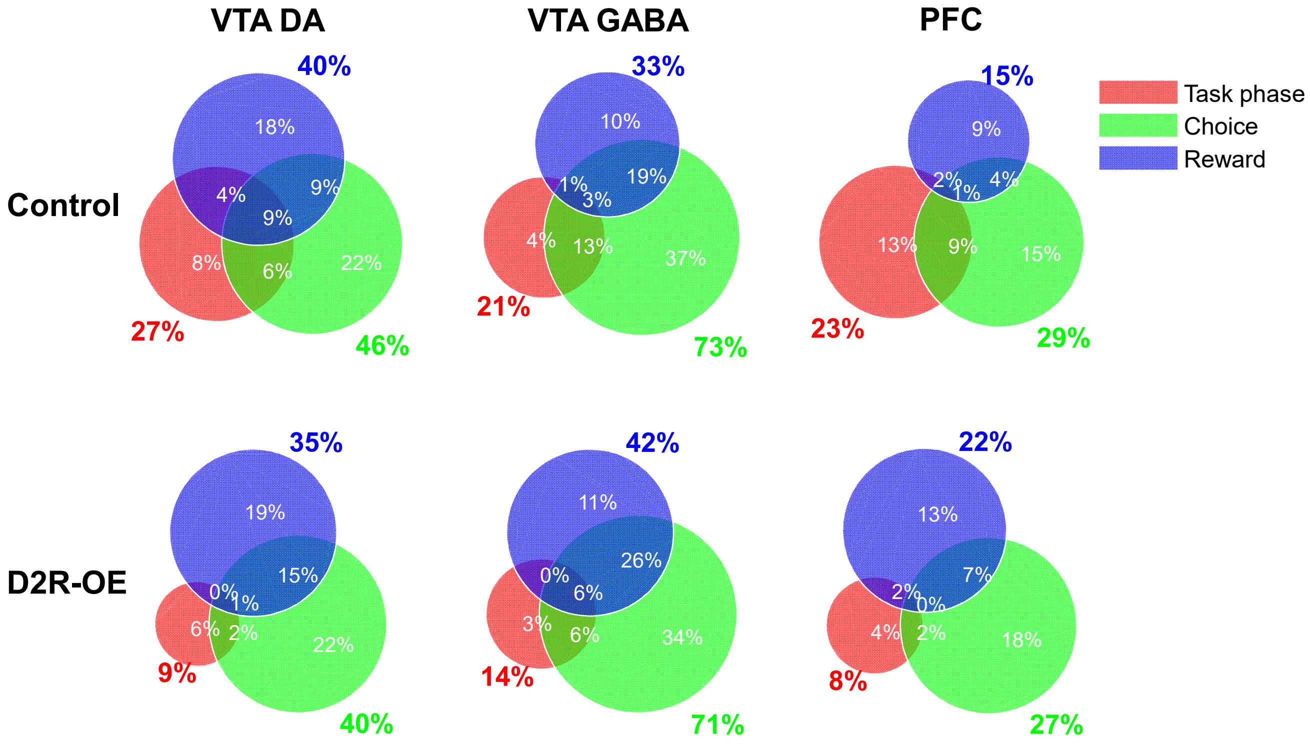

Supplementary Figure 10. Overlap of task-related response categories. Each Venn diagram indicates the proportion of VTA and PFC neurons in control and D2R-OE mice that were modulated exclusively by task-phase, choice or reward (non-overlapping areas) as well as the proportion of neurons modulated by more than one response variable (indicated by overlapping areas). Percentages in color represent the total percentage of neurons modulated by each response variable. N values are as in Figures 6 and 7.
